# Effect of local papaverine on arteriovenous fistula maturation in patients with end-stage renal disease

**DOI:** 10.1590/2175-8239-JBN-2018-0170

**Published:** 2019-04-11

**Authors:** Reza Manani, Gholamreza Kazemzadeh, Ali Saberi, Fatemeh Sadeghipour, Asghar Rahmani

**Affiliations:** 1 Zanjan University of Medical Science Fellowship of Vascular Surgery School of Medicine Zanjan Iran Zanjan University of Medical Science, Fellowship of Vascular Surgery, School of Medicine, Zanjan, Iran.; 2 Mashhad University of Medical Science School of Medicine Mashhad Iran Mashhad University of Medical Science, School of Medicine, Mashhad, Iran.; 3 Mashhad University of Medical Science Vascular Surgery Research Center Mashhad Iran Mashhad University of Medical Science, Vascular Surgery Research Center, Mashhad, Iran; 4 Ilam University of Medical Sciences Ilam Iran Ilam University of Medical Sciences, Iran, Ilam

**Keywords:** Arteriovenous Fistula, Papaverine, Kidney Failure, Chronic

## Abstract

**Background:**

Arteriovenous fistula (AVF) maturation is one of the main concerns in patients with end-stage renal disease (ESRD) and finding a strategy for increasing success rate and accelerating fistula maturation is valuable. The aim of this study was to evaluate the effects of papaverine injection on AVF maturation and success rate.

**Method:**

This study was a randomized clinical trial that involved 110 patients with ESRD that were referred for AVF construction. Patients were allocated in papaverine group and control group with block randomization according to age and sex. In the case group, papaverine (0.1 or 0.2 cc) was injected locally within the subadventitia of artery and vein after proximal and distal control during AVF construction and in the control group, AVF construction was done routinely without papaverine injection.

**Results:**

Maturation time in case and control groups was 37.94 ± 11.49 and 44.23 ± 9.57 days, respectively (p=0.004). Hematoma was not seen in the case group but occurred in one patient in the control group. One patient of the case group developed venous hypertension. Four functional fistulas, 1 (1.8%) in the case group and 3 (5.5%) in the control group, failed to mature (p=0.618). Maturation rate did not differ between the two groups statistically (p=0.101).

**Conclusion:**

Local papaverine injection increased vessel diameter and blood flow, increasing shearing stress in both arterial and venous segment of recently created AVF. In this way, papaverine probably can decrease AVF maturation time without an increase in complications.

## INTRODUCTION

According to epidemiological studies, about 10% of patients with chronic renal failure (CRF) undergo renal transplant and the other 90% that do not receive a renal transplantation must remain in dialysis therapy (hemodialysis or peritoneal dialysis).^1^ End-stage renal disease (ESRD) is an important health problem with significant morbidity, mortality, and socioeconomic effects in the community.^2^ Today, arteriovenous fistula (AVF) is the preferable vascular access in patients who undergo hemodialysis.^3^ Due to the high number of patients with ESRD, finding a way to increase the success rate of native AVF is of great value and improves the quality and quantity of life of these patients, reducing medical expenses.^4^

Papaverine is an inexpensive, readily available drug with few side effects, and it is an opioid derivative, which relaxes the smooth muscles of the vessel wall. This effect is due to inhibition of phosphodiesterase and increase of cyclic adenosine monophosphate (C-AMP).^5^^,^^14^ The half-life of this drug is ninety minutes and the drug is metabolized in the liver. Hemodialysis causes the drug's clearance. There is no age limitation for its prescription, and its side effects, like bradycardia and apnea, are seen in systemic routes of consumption (intravenous or oral); no serious side effect is seen in local administration.

So far, there are few clinical trials evaluating the clinical effects of papaverine on AVF. According to those few studies, the local application of papaverine resulted in a reduced rate of early thrombosis from 12 to 5.5%.^6^^,^^13^ We searched for clinical trials that investigated the effects of local papaverine use on distal upper extremity AVF success rate and its early complication and we did not find any research. Therefore, the aim of this study was to investigate the role of local papaverine administration in reducing the complications and improving the success rate of distal AVF.

## MATERIALS AND METHODS

### Study Design and Population

This study was a prospective clinical trial performed in the vascular surgery clinic of Imam Reza's Hospital of Mashhad University of Medical Sciences, Mashhad, Iran. The study involved 110 patients with ESRD who were referred by nephrologists to hemodialysis. The inclusion criteria were: patients with ESRD who needed hemodialysis and were suitable for AVF construction in distal upper limbs (snuffbox and distal forearm) based on physical examination and Doppler sonography. Exclusion criteria were: diabetic patients, patients with systolic blood pressure <120 mm/hg during operation, previous history of AVF construction on both upper limbs, and allergy to papaverine. After complete description of this procedure and information on papaverine (side effects and benefits), informed consent was obtained from patients. After matching for age and sex, patients were divided into two equal groups according to block randomization with random block size; Papaverine (case) group or control group. There were blocks of even numbers (2, 4 and 6) of subjects allocated to Papaverine treatment and control group and in all blocks the subjects were distributed evenly and randomly. For example, in a 6-person block, 3 subjects were allocated to papaverine group and 3 subjects in the control group. The order of treatment of each group member was chosen randomly. The blocks were also chosen randomly. This was a triple blind study.

This study was approved by the local ethics committee and the proposal code is 910600. Also, this study is registered in the Iranian Registry of Clinical Trials with code IRCT20171023036953N2. All participating patients signed the informed consent form. This thesis was sponsored by research deputy of Mashhad University of Medical Science, Iran.

### Intervention

The control group underwent AVF construction by the conventional method without using papaverine and the case group underwent construction of autogenous AVF access with local papaverine (Exir Medical Inc., Iran). Factors influencing vascular access selection included: artery diameter >2.0 mm, vein diameter >3.0 mm, difference in systolic blood pressure between the two upper limbs less than 20 mm/Hg, proper proximity between artery and vein, and complete palmar arch. The absence of signs of central vein stenosis, segmental stricture or occlusion in the superficial vein, ischemia in upper limb or scars or wounds at the site of AVF construction in physical examinations were confirmed by a surgeon. The non-dominant hand was preferred for AVF construction. Before surgery, sonography was performed for evaluation of access flow rate and vein diameter by a sonographer.

**Figure 1:**
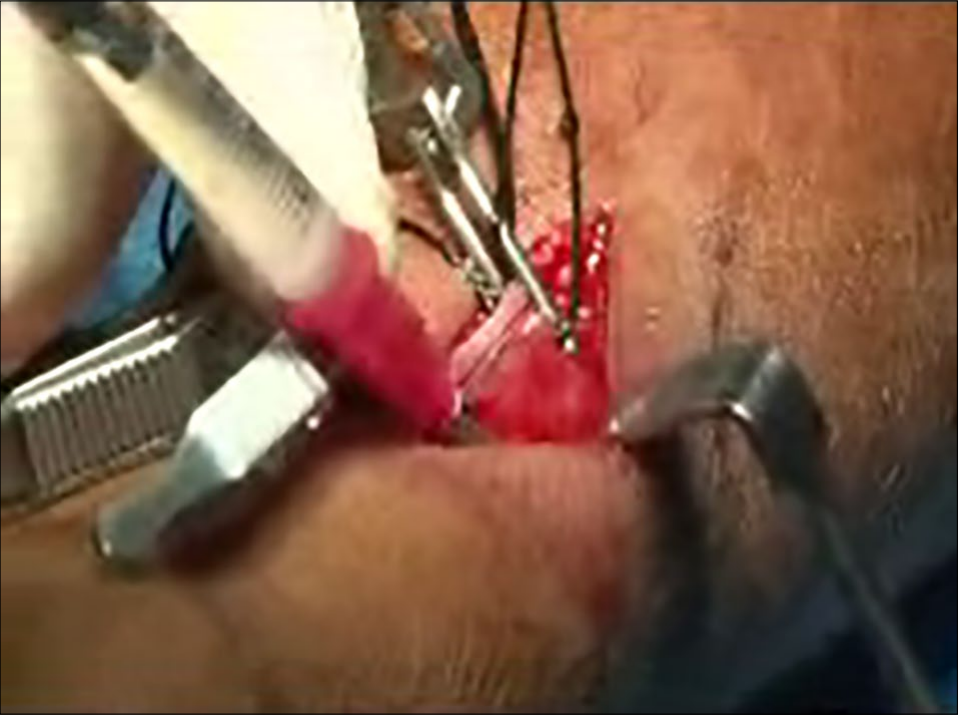
Image of Papaverine subadventitia injection procedure conducted in this study.

All patients underwent surgery with the same surgical team and one surgeon. Under local anesthesia with lidocaine 1% (Exir Medical Inc., Iran), we performed a longitudinal incision about 3-5 cm with sharp dissection and minimal manipulation of the vessels. We did not use mechanical dilation of vessels and vascular branches were not ligated when possible to avoid under tension anastomosis. During the operation, we did not use heparin. Artery and vein were occluded with microvascular clamps. The amount of 0.1 to 0.2 cc papaverine (papaverine HCl 40 mg/mL, Exir Medical Inc., Iran) was injected with 30-gauge syringe within the sub adventitia of artery and vein (Figure 1). The end-to-side anastomosis was performed using a running 7-0 monofilament vascular suture for distal forearm AVFs and side-to-side anastomosis for snuffbox AVFs in the same manner. Patients were visited weekly for 3 weeks and then monthly for 3 months [on days 7, 14, 21, 30, 60, 90] or until fistula maturation. When fistula matured clinically, a second Doppler sonography was performed to confirm maturation based on the KDOQI guidelines 7. If fistula maturation was not obtained until 3 months, the patient was excluded from the study. Patients were evaluated for surgical site complications (hematoma, seroma and infection), early thrombosis, venous hypertension, steal syndrome, and neuropathy at every post-operation visit.

### Statistical analysis

The quantitative and qualitative data are presented as the mean ± standard deviation (SD) and frequency, respectively. All statistical analysis of the data was performed by SPSS 19 (SPSS Inc, Chicago, USA) software. A p-value <0.05 was considered significant.

## RESULTS

**Figure 2:**
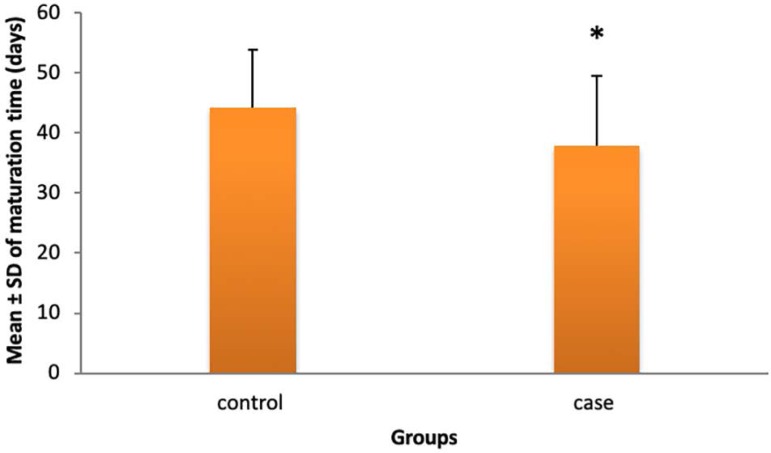
Average maturation time in Papaverine and control groups in days.

**Figure 3:**
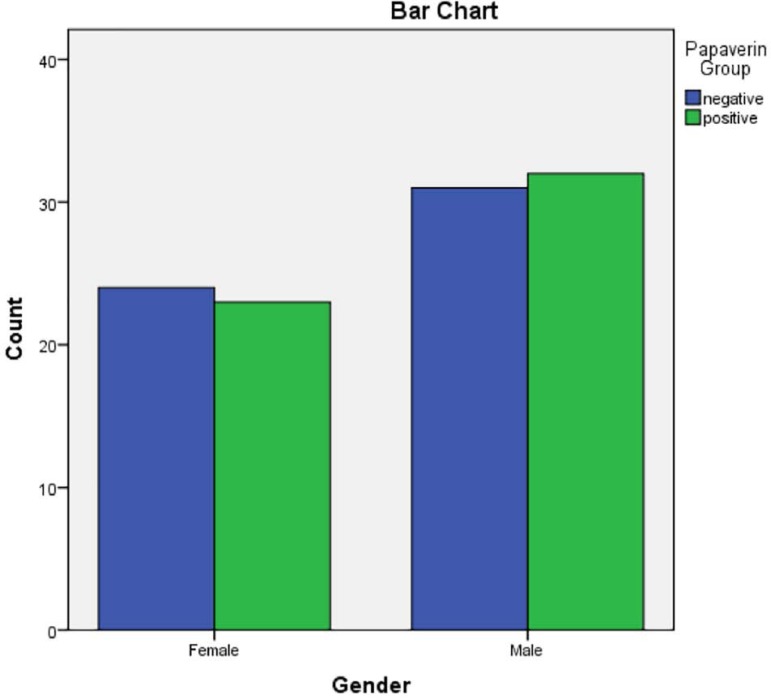
Gender difference of participating patients.

**Figure 4:**
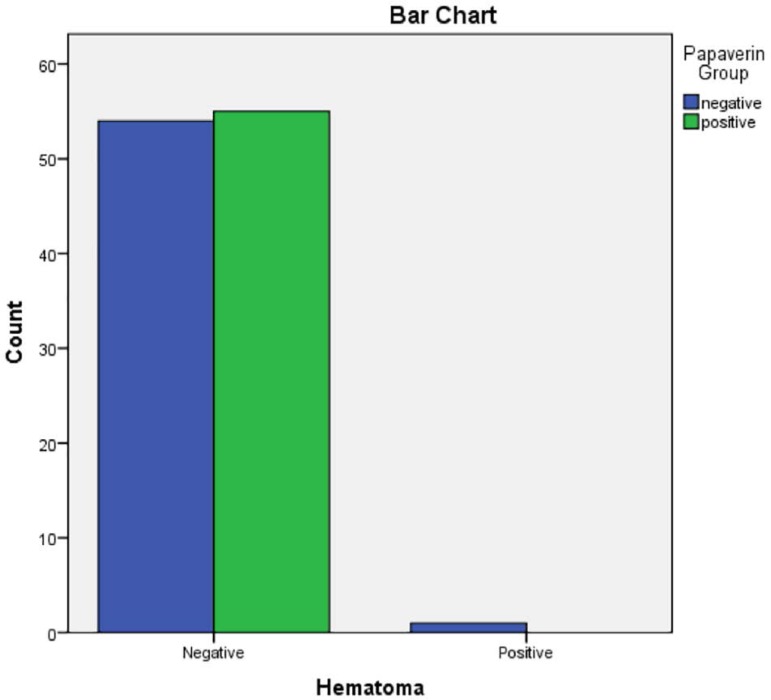
Presence of hematoma in participating patients.

**Figure 5:**
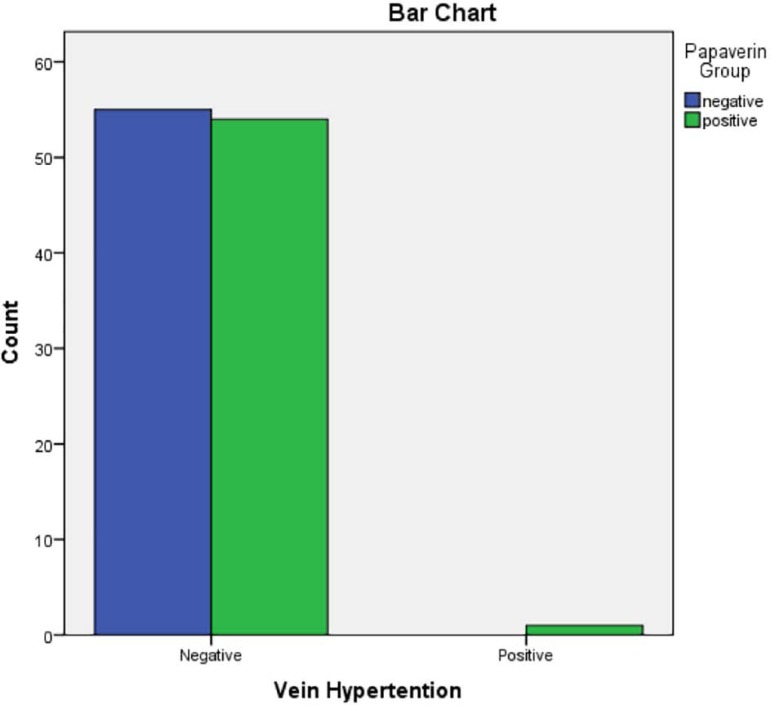
Presence of venous hypertension in participating patients.

**Figure 6:**
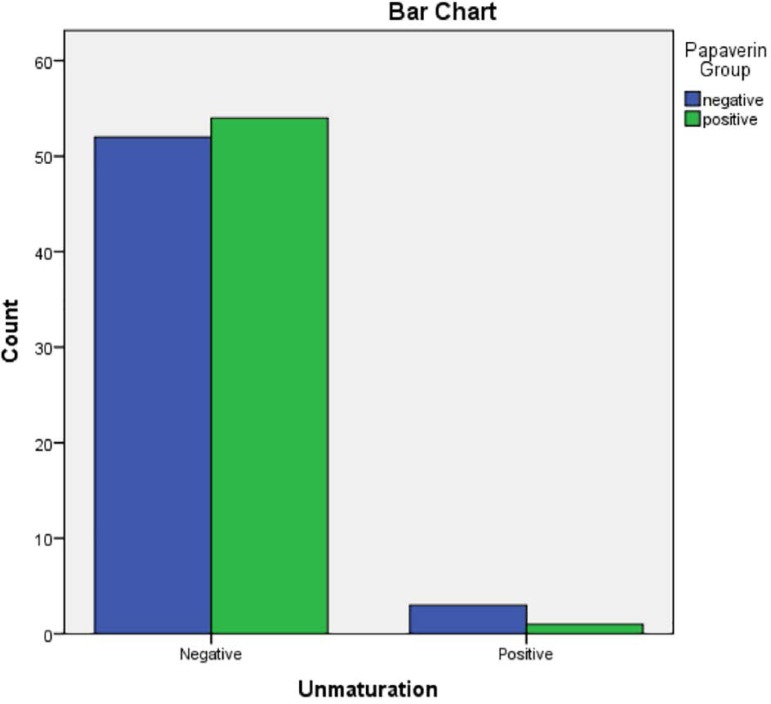
Non-maturation comparison.

Patients were allocated into 4 categories based on age and sex. Group A: <15 years old, group B: 15-35 years old, group C: 35-50 years old, group D: >50 years old. Overall, 110 patients were enrolled in this study. The papaverine group consisted of 23 (42%) women and 32 (58%) men and the control group consisted of 24 (44%) women and 31 (56%) men. Gender difference was not seen between the groups (P=0.847) (Table 1 and Figure 2). The mean age of the papaverine group was 50.96 years (SD=11.86) and the control group, 49.21 years (SD=11.97 years) (Table 2). The age difference between the two groups was not significant (p=0.443). Hematoma was not seen in the papaverine group and occurred in one patient in the control group (Table 3, Figure 3). One patient of the papaverine group developed venous hypertension (Table 4) (Figure 4). Early thrombosis was seen in 2 (3.6%) patients of the papaverine group and 5 (9.1%) patients of the control group, which was not statistically significant (p=0.438). Four functional fistulas, 1 (1.8%) in the papaverine group and 3 (5.5%) in the control group, failed to mature (p=0.618) (Figure 5). Maturation time was 37.94 days (SD=11.497) in the papaverine group and 44.23 days (SD=9.572) in the control group, which was statistically significant (p=0.004) (Figure 6). Maturation rate did not differ between the two groups statically (p=0.101).

**Table 1:** Gender difference in participating patients

Papaverine Group			Frequency	Percent	Valid Percent
Negative	Valid	Female	24	43.6	43.6
		Male	31	56.4	56.4
		Total	55	100.0	100.0
Positive	Valid	Female	23	41.8	41.8
		Male	32	58.2	58.2
		Total	55	100.0	100.0
Chi-square tests					
	Value	df	Asymptotic Significance (2-sided)	Exact Sig. (2-sided)	Exact Sig. (1-sided)
Pearson Chi-Square	0.037^a^	1	0.847		
Continuity Correction^b^	0.000	1	1.000		
Likelihood Ratio	0.037	1	0.847		
Fisher's Exact Test				1.000	0.500
Linear-by-Linear Association	0.037	1	0.848		
N of Valid Cases	110				

a 0 cells (.0%) have expected count less than 5. The minimum expected count is 23.50.

b Computed only for a 2x2 table

**Table 2:** Presence of hematoma in participating patients

Papaverine Group			Frequency	Percent	Valid Percent
Negative	Valid	Negative	54	98.2	98.2
		Positive	1	1.8	1.8
		Total	55	100.0	100.0
Positive	Valid	Negative	55	100.0	100.0
Hematoma * Papaverine Group Crosstabulation					
			Papaverine Group		Total
			Negative	Positive	
Hematoma	Negative	Count	54	55	109
		Expected Count	54.5	54.5	109.0
	Positive	Count	1	0	1
		Expected Count	.5	.5	1.0
Total		Count	55	55	110
		Expected Count	55.0	55.0	110.0
Chi-Square Tests					
	Value	df	Asymptotic Significance (2-sided)	Exact Sig. (2-sided)	Exact Sig. (1-sided)
Pearson Chi-Square	1.009^a^	1	.315		
Continuity Correction^b^	.000	1	1.000		
Likelihood Ratio	1.395	1	.237		
Fisher's Exact Test				1.000	.500^c^
Linear-by-Linear Association	1.000	1	.317		
N of Valid Cases	110				

a 2 cells (50.0%) have expected count less than 5. The minimum expected count is .50.

b Computed only for a 2x2 table.

c There is no significant difference in the presence of hematoma between the two groups.

**Table 3:** Presence of venous hypertension in participating patients

Papaverine Group			Frequency	Percent	Valid Percent	Cumulative Percent
Negative	Valid	Negative	55	100.0	100.0	100.0
Positive	Valid	Negative	54	98.2	98.2	98.2
		Positive	1	1.8	1.8	100.0
		Total	55	100.0	100.0	
Venous Hypertention * Papaverine group crosstabulation						
				Papaverine Group		
				Negative	Positive	Total
Venous Hypertention		Negative	Count	55	54	109
			Expected Count	54.5	54.5	109.0
		Positive	Count	0	1	1
			Expected Count	.5	.5	1.0
Total			Count	55	55	110
			Expected Count	55.0	55.0	110.0
Chi-Square tests						
		Value	df	Asymptotic Significance (2-sided)	Exact Sig. (2-sided)	Exact Sig. (1-sided)
Pearson Chi-Square		1.009^a^	1	.315		
Continuity Correction^b^		0.000	1	1.000		
Likelihood Ratio		1.395	1	.237		
Fisher's Exact Test				1.000		.500^c^
Linear-by-Linear Association		.000	1	.317		
N of Valid Cases		110				

a 2 cells (50.0%) have expected count less than 5. The minimum expected count is .50.

b Computed only for a 2x2 table.

c There is no significant difference in venous hypertension between the two groups.

**Tabela 4:** Non-maturation comparison

Papaverine Group			Frequency	Percent	Valid Percent	Cumulative Percent
Negative	Valid	Negative	52	94.5	94.5	94.5
		Positive	3	5.5	5.5	100.0
		Total	55	100.0	100.0	
Positive	Valid	Negative	54	98.2	98.2	98.2
		Positive	1	1.8	1.8	100.0
		Total	55	100.0	100.0	
Non maturation * Papaverine Group Crosstabulation						
				Papaverine Group		
				negative	positive	Total
Unmaturation		Negative	Count	52	54	106
			Expected Count	53.0	53.0	106.0
		Positive	Count	3	1	4
			Expected Count	2.0	2.0	4.0
Total			Count	55	55	110
			Expected Count	55.0	55.0	110.0
Chi-Square Tests						
	Value	df	Asymptotic Significance (2-sided)	Exact Sig. (2-sided)	Exact Sig. (1-sided)	
Pearson Chi-Square	1.038^a^	1	.308			
Continuity Correction^b^	.259	1	.611			
Likelihood Ratio	1.084	1	.298			
Fisher's Exact Test				.618	.309^c^	
Linear-by-Linear Association	1.028	1	.311			
N of Valid Cases	110					

a 2 cells (50.0%) have expected count less than 5. The minimum expected count is 2.00.

b Computed only for a 2x2 table.

c There is no significant difference in maturation occurrence between the two groups.

## DISCUSSION

After AVF creation, shearing stress should be raised in the venous segment. Vessel diameter is one of the important factors in shearing stress 6^,^^19^^,^^20^. Saucy et al. showed that blood flow <120 cc/min is a prognostic factor in early AVF failure 8, so injection of papaverine into the vessel wall during the procedure leads to relaxation of smooth muscle cells of the vessels and increase vessel diameter and blood flow, which can accelerate AVF maturation.

Many studies have focused on the effect of antiplatelet agents such as ASA, dipyridamole, clopidogrel, and ticlopidine due to the antithrombotic effects of these drugs.^15^^,^^17^^,^^18^ One randomized double-blind study showed that dipyridamole with aspirin caused mild but significant increase of unassisted primary patency in one year (CHR: 0.82; CI:0.68-0.98; P=0.03) but did not have a significant effect on cumulative survival.^9^ Four studies evaluated the effect of dipyridamole on AVF and showed that the drug decreased thrombosis in both AVF and AVG during a short period of time but studies limitations were low sample size, short follow up time and failure to report confounding variables.^10^

The effect of clopidogrel was evaluated in three studies. In the latest study, carried out by dialysis access consortium (DAC), AVF thrombosis rate was evaluated for six weeks after the procedure. Eight hundred and seventy-seven patients were randomly allocated to clopidogrel or placebo. The study showed that, although there was a decrease in AVF obstruction for six weeks (relative risk=0.63), a significant effect was not observed on the secondary outcome (fistula usability).^11^

Lyme et al. presented the results of 411 AVF fistula procedures. They used local papaverine and mechanical dilatation if vasospasm occurred during the procedure. The authors showed that the rate of early thrombosis was 5.98%, which was less than values reported in the literature, and they attributed the results to mechanical dilatation with a probe to relieve arterial and venous spasm and use of local papaverine.^12^

Because of intimal injury from mechanical probing dilatation, we used only local papaverine injection into the arterial and venous wall. In our study, there was no statistically significant difference between the two groups in terms of maturation rate and post-operation complications but maturation time in the papaverine group was significantly lower than in the control group (37.94 days vs 44.23 days, P=0.004). In our study, maturation success rate was higher than other studies, which could be due to the papaverine vascular effect (vasodilatation that leads to early maturation). In addition, the selection of patients that participated in this study may have affected the results; for example, the failure to maturation in diabetic patients, which were not enrolled in our study, is higher than non-diabetic patients.

Finally, due to the papaverine safe pharmacologic profile, its low price, and easy injection technique, it can be considered a suitable drug for acceleration of maturation after AVF construction. However, further studies with larger sample size are needed to investigate in detail the local papaverine effects on AVF maturation in patients with ESRD.
