# Erratum

**DOI:** 10.1590/2175-8239-JBN-2018-0170er

**Published:** 2019-11-04

In the article “Effect of local papaverine on arteriovenous fistula maturation in patients with end-stage renal disease”, with the DOI http://dx.doi.org/10.1590/2175-8239-jbn-2018-0170, published in Brazilian Journal of Nephrology, vol. 41, n. 2, Apr./June 2019, Epub Apr 11, 2019:

Where it was written:

Reza Manani^1^

Gholamreza Kazemzadeh^2^

Ali Saberi^2^

Fatemeh Sadeghipour^3^

Asghar Rahmani^4^ 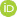


^1^Zanjan University of Medical Science, Fellowship of Vascular Surgery, School of Medicine, Zanjan, Iran.

^2^Mashhad University of Medical Science, School of Medicine, Mashhad, Iran.

^3^Mashhad University of Medical Science, Vascular Surgery Research Center, Mashhad, Iran.

^4^Ilam University of Medical Sciences, Iran, Ilam.

Should read:

Gholamhosein Kazemzadeh^1^

Ali Saberi^1^

Reza Mannani^2^

Fatemeh Sadeghipour^1^

Asghar Rahmani^3^ 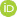


^1^Mashhad University of Medical Sciences, Vascular and Endovascular Surgery Research Center, Mashhad, Iran.

^2^Zanjan University of Medical Sciences, School of Medicine, Zanjan, Iran.

^3^Ilam University of Medical Sciences, School of Medicine, Ilam, Iran.

